# Submental Intubation in Maxillofacial Procedures: A More Desired Approach Than Nasotracheal Intubation and Tracheostomy

**DOI:** 10.7759/cureus.27475

**Published:** 2022-07-30

**Authors:** Kerry-Ann D Williams, Maha Tariq, Maitri V Acharekar, Sara Elena Guerrero Saldivia, Sumedha Unnikrishnan, Yeny Y Chavarria, Adebisi O Akindele, Ana P Jalkh, Aziza K Eastmond, Chaitra Shetty, Syed Muhammad Hannan Ali Rizvi, Joudi Sharaf, Lubna Mohammed

**Affiliations:** 1 Research, California Institute of Behavioral Neurosciences & Psychology, Fairfield, USA; 2 Internal Medicine, California Institute of Behavioral Neurosciences & Psychology, Fairfield, USA

**Keywords:** skull fracture procedures, anesthesia, throat, emergency airway, intubate, tracheostomy, endotracheal intubation, nasal cavity, airway management, nasotracheal intubation

## Abstract

To achieve adequate airway management in maxillofacial procedures, the right intubation technique should be employed. This is because the surgeons and the anesthesiologists will need to work in the same surgical field to ensure a successful procedure. The type of intubation method used can either complicate either's role or pose some difficulties in the surgery itself. Nasotracheal intubation and orotracheal intubation may often be contraindicated in different types of maxillofacial surgeries and due to the complications associated with a tracheostomy, this method is often utilized as a last resort. Submental intubation has become very popular and favored alternative and has been associated with fewer complications. This literature review was conducted to explore the indications, complications, and contraindications of the different intubation methods. Sources were gathered from PubMed Central, PubMed, and Google scholar and included articles published between 2012 and 2022. A mix of literature reviews, case base studies, retrospective studies, prospective studies, and a few systematic reviews were examined. It was found that the use of submental intubation was preferred due to its less invasive nature, minimal intraoperatively and postoperatively complications, and greater patient compliance compared to tracheostomy. In addition, it is the best method when Nasotracheal intubation is contraindicated.

## Introduction and background

During maxillofacial surgeries, both surgeons and anesthesiologists must work in the same operational field. Understandably this has presented the specialists with some difficulty as airway management and anesthesia maintenance are just as vital as the surgical procedure [1]. The success of such a delicate challenge requires supreme knowledge, skills, and collaboration from both specialists to ensure that patient safety is met intraoperatively as well as postoperatively and thereby decreasing associated morbidity and possible mortality [2]. Several techniques can be employed by anesthesiologists in establishing a safe airway during maxillofacial procedures. It is their responsibility to ensure that the best-suited technique that reduces peri and postoperative complications, is utilized. Nasotracheal intubation is the passage of an endotracheal tube through the naris into the nasopharynx and the trachea. It is commonly used for dental, oropharyngeal, and maxillofacial procedures [3]. Nasotracheal intubation is associated with increased complications in patients with skull base fractures, some of which are cerebrospinal fluid leakage and meningitis. Tracheostomy is a surgical procedure that involves the creation of a new respiratory channel in the cervical trachea [4]. It is a reasonable substitute for nasotracheal intubation, but it also poses some risks as it requires attentive postoperatively care and lack thereof will lead to a rise in morbidity and rates of complications. Therefore, it is important to consider and rule out contraindications for the utilization of nasotracheal intubation and tracheostomy in maxillofacial surgical procedures [5].

Surgeries that pose an increased risk of adverse events when nasotracheal intubation is employed are comminuted midfacial fractures, skull base fractures, and naso-orbital-ethmoidal reconstructive procedures. There is extreme difficulty in the passage of a nasotracheal tube as comminuted fractures impede the smooth passage of the tube and progression will create more harm to the patient. There will also be limited reconstructive space for the surgeons due to the tube's occupancy [6]. A tracheostomy tube is normally placed between the second and fourth tracheal rings. Access is gained after a cutaneous incision is made and blunt dissection is done through muscles, sheaths, and fascia until the trachea is reached. This technique is associated with perioperative complications such as incidental injury to surrounding structures of the cervical neck including cartilages, muscles, and membranes. Bleeding from injury to blood vessels and even esophageal laceration may occur. Tracheostomy is associated with a 1% perioperative mortality risk [4].

A safer technique is to be desired for maxillofacial and skull base surgical procedures. One that is associated with less morbidity and mortality and is relatively easy to perform with proper training. A technique that will provide both the resuscitating anesthesiologists and the maxillofacial surgeons with the ability to effectively perform their respective duties in the operating room. In 1986, Hermandez Altemic pioneered the use of submental intubation as a more desired technique of establishing excellent airway control during surgeries involving craniomaxillofacial injuries while also providing access to the buccal and nasal cavity via a tube passing through the submandibular region of the mouth [4,7,8]. Both maxillofacial surgeons and anesthesiologists can boast about the minimal complications associated with this technique and their preference for its use over tracheostomy and nasotracheal intubation. This review paper aims to elaborate on and summarize the uses, complications, and contraindications associated with the different forms of intubations and highlight the use of submental intubation as the most suitable alternative in specific surgeries.

## Review

In patients undergoing repair of maxillofacial fractures, thorough discussions are necessary between the Surgeons and the Anesthesiologists about the most appropriate route for intubation. The operative field is shared by both specialists and inadequate access by either can lead to suboptimal patient management. In cases where the surgical field will be compromised by an endotracheal tube, it is important to decide between nasotracheal intubation, tracheostomy, and submental intubation [9]. Airway management in these patients may present with some amount of difficulties as, depending on the case and extent of craniomaxillofacial damage, both orotracheal and Nasotracheal intubation may be contraindicated [10]. The morbidity and mortality are significantly increased when ventilation is compromised. Gupta et al. reported that among 2,594 patients with maxillofacial trauma, poor airway protection and inadequate ventilation accounted for 16% of deaths. While it is understandable that difficulties can arise in mask ventilation in this patient population, the most suitable means of securing and maintaining the airway must be sought and implemented on a case-by-case basis. Difficulties due to bone distortion, facial edema, asymmetry, occlusion by blood, oral secretions, and emesis were encountered [9].

Nasotracheal intubation indications and associated morbidity and mortality 

According to Park et al., nasotracheal intubation is normally indicated in patients who are undergoing maxillofacial, intranasal, oropharyngeal, mandibular surgery, dental procedures, and surgeries involving the cervical spine where there may be cervical spine degenerative diseases and instability. Other indications and contraindications of nasotracheal intubations explored in some of the cases and articles reviewed are tabulated in Table 1 [11].

**Table 1:** Indications and contraindications of nasotracheal intubation Adapted with permission from The Korean Society of Anesthesiologists 2021 [11].

Indications of nasotracheal intubation	Contraindications of nasotracheal intubation
Mandibular surgery	Naso-ethmoidal fractures
Maxillofacial surgery	Fractures of anterior and middle cranial fossa
Intranasal surgery	Skull base fractures
Oropharyngeal surgery	Recurrent epistaxis
Dental surgery	Nasal foreign bodies
Degenerative diseases and instability of the Cervical spine.	Nasal polyps
Trismus leading to restricted mouth opening	Coagulopathy

Nasotracheal intubation is not ideal in patients who present with fractures of the base of the skull and fractures of the nasal bones. These types of injuries may also be present in patients experiencing traumatic brain injury. Trauma can lead to alteration of the normal anatomy of the airway and will pose additional difficulty for the insertion of a Nasotracheal tube. Performing the procedure in these patients will lead to severe adverse outcomes especially if the tube was inserted blindly. While Fiberoptic bronchoscopy-guided intubation or a video laryngoscopy with the right forceps can be used to guide the endotracheal tube appropriately and may contribute to reducing morbidity, the absolute contraindications mention in Table 1 should be considered and nasotracheal intubation is to be avoided [10-13]. Consequences may involve intracranial penetration of the tube which may result in cerebrospinal spinal fluid leakage as well as infection of the epidural space. This is more than likely in the cases of fractures of the base of the skull [8,14].

When nasotracheal intubation is indicated, it does not mean a smooth passage of the tube will ensue. Edema of the nasal passage and deviation of the nasal septum may still pose a challenge for passage of the Endotracheal tube and complications such as transfer of infectious agents from the nose to the bronchial tree, retropharyngeal perforation, and hematoma can develop. Considerations should be done in patients who require prolonged intubation as well. These patients are prone to developing nasal pressure sores and deformed nasal anatomy [10,11].

Tracheostomy indications and associated complications

Tracheostomy is the definitive airway; it is a traditional approach to airway management that is usually considered to be safe. It is indicated in cases with prolonged ventilation and where submental intubation, endotracheal and nasotracheal intubation is contraindicated [9,10,15]. It is often used as a last resort. Generally, the use of tracheostomy may be preferred because the patient may experience less discomfort during and after the procedure. There is also the fact that a tracheostomy is of low and easy maintenance as the tracheal fluids can be aspirated through the tracheal opening and oral cleanliness can be well maintained. Also reinserting the tracheostomy tube is generally a simple and easy task. Regardless, tracheostomy is associated with significant morbidity and the complications that can arise can be characterized by immediate and late complications. Nerve injury, bleeding, and the development of subcutaneous emphysema or a pneumomediastinum can be seen in the early periods of performing the procedure. According to Szantyr et al. stimulation of the vagus nerve can lead to cardiac arrest in the early stages of insertion. It was also reported that an increase in carbon dioxide levels may develop which can lead to acidemia which, if severe enough, may result in post hypercarbic shock and pulmonary edema due to impaired alveolar ventilation [10]. Air embolism can be added to the list of general and early complications of the procedure [16,17]. While complications such as tracheal stenosis, tube blockage, respiratory infection, development of a tracheoesophageal fistula, voice changes, tracheal granuloma, unfavorable scar tissue formation, and deformity may present in the later periods following the procedure [9-11,18-22]. Reportedly early complications of tracheostomy had an incidence of 6%-8% while the incidence of late complications was 60% [10]. To avoid these complications, submental intubation is by far more desired as its morbidity is low in comparison to that of a tracheostomy [15,21]. Table 2 lists the indications and complications of tracheostomy [17].

**Table 2:** Indications and morbidity associated complications of tracheostomy Information was gathered with permission from the Tehran University of Medical Science 2017 [17].

Indications of Tracheostomy	Morbidity/Complications of Tracheostomy
Patients requiring prolonged ventilation	Cardiac Arrest, air embolism, Pneumomediastinum, Pneumothorax
Multi-trauma patients	Damage to the Recurrent Laryngeal nerve and Thyroid gland
Severe neurological Damage	Subcutaneous Emphysema
Major Thoracic trauma	Intraoperative damage by electrocautery-associated fires
Patients requiring repeated operations	Laryngeal and tracheal strictures and stenosis, Fistula formation, hemorrhage
Patients with severe damage to the floor of the mouth	Respiratory infections, dysphagia, Excessive scar tissue formation, cosmetic deformities
Severe bleeding in Oral Cavity	Tracheomalacia, voice alteration, and tracheal granuloma formation

Indications for submental intubation

According to Jundt et al.'s systematic review, the main and most common indications for submental intubation were jaw fractures resulting from trauma as well as simple jaw fractures that required the restoration of a functional airway. These cases were of patients with nasal fractures, naso-orbital-ethmoidal (NOE), and skull base fractures [23]. Cheong et al. reported on a case of an 18-year-old man who presented with an NOE and required intraoperative assessment of dental occlusion and open reduction and internal fixation of the fracture. Submental intubation was the chosen method of intubation for the surgery as nasotracheal intubation was not indicated due to his fracture type and endotracheal intubation could not be done due to the required dental occlusion. He also reported a second case of a 48-year-old man who presented with a bilateral orbital wall, maxillary fractures, and associated skull base fractures. This patient previously had an epidural hematoma evacuated eleven days prior and his mental status was alert enough after the procedure to avoid intubation therefore submental intubation was chosen over a tracheostomy. In both cases, long-term ventilatory support was not required [5].

Other indications were due to facial cosmetic surgery and rhinoplasty. Patients presenting with congenital defects or deformities of the nose and throat were also considered. In all these cases the other forms of intubation mentioned above were not indicated [13,15,23,24]. Indications, as well as contraindications, have been tabulated in Table 3 [24].

**Table 3:** Indications and contraindications of submental intubation Information gathered with permission from The Korean Association of Oral and Maxillofacial Surgeons [24].

Indications	Contraindications
Contraindications to nasotracheal intubation: Epistaxis, CSF fluid leakage, rhinorrhea	Prolong ventilation required
Pan facial trauma	Severe neurological deficits
Basal bone fractures	Repeated surgeries required
Orthognathic surgery +Rhinoplasty	Mandibular surgeries
Craniomaxillary surgery	Damage to the floor of the mouth
	Patient refusal

**Figure 1:**
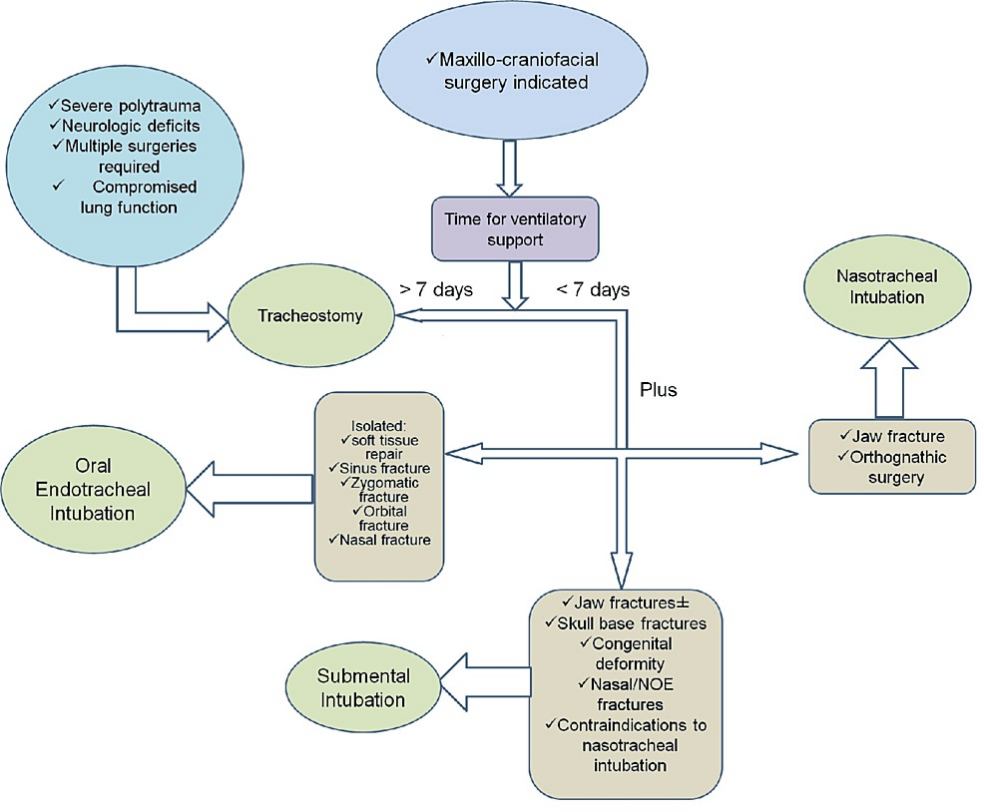
Algorithmic presentation of the indications of the different forms of intubation Recreated from International Journal of Oral and Maxillofacial Surgery, 2012 [23].

One can follow an algorithm or guide to the different forms of intubation. If craniofacial surgery is required and ventilatory support is needed for seven days or more a tracheostomy is considered. The additional presence of neurological deficits, anticipation of additional surgeries, and a compromised state of the lungs will also warrant the use of a tracheostomy. If ventilatory support is required for seven days or less and the case is a single and remote fracture of the nose, sinus, or orbits including regions of the maxillary zygomatic complex, a simple oral endotracheal intubation can be utilized. Nasotracheal intubation is considered in the cases of orthognathic, and jaw fracture surgeries that require less than seven days of mechanical ventilation. Submental intubation is also possible but may not be done due to aesthetic reasons as there are varied reports of scarring and some patients may not be willing to tolerate this complication of submental intubation. Mechanical ventilation for seven days or less is an ideal time for the use of submental intubation for patients with nasal fractures, NOE fractures, and skull base fractures. This algorithm can be depicted in Figure 1 [23].

Steps for the procedure

**Figure 2:**
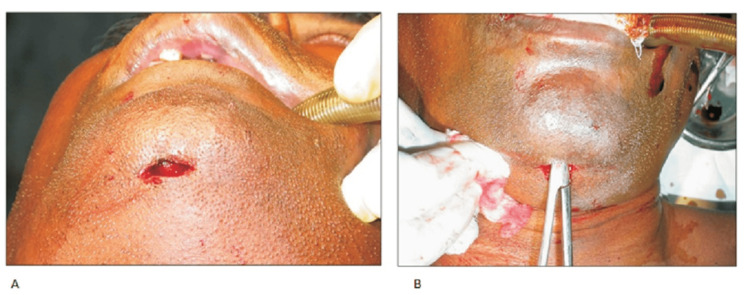
(A) Transverse incision made for submental intubation. (B) Hemostat placed Images obtained from The Korean Association of Oral and Maxillofacial Surgeons. Copyright © 2016 The Korean Association of Oral and Maxillofacial Surgeons. All rights reserved. License link (http://creativecommons.org/licenses/by-nc/4.0/) [7].

Submental intubation allows for the passage of an endotracheal tube through the anterior floor of the oral cavity. This allows for adequate surgical access to the regions of the mouth and the nasal pyramid with minimal to no hindrance in airflow to the lungs. According to Kumar et al., after proper aseptic procedures are adhered to, the procedure is first carried out by performing intubation via the orotracheal route under general anesthesia. When induction of anesthesia is achieved, conversion to the submental route of intubation ensues. As shown in Figure 2A, this is done by making a 1-cm transverse incision in the midline of the face and below the lower border of the mandible in a region known as the submental crease. While ensuring that the tongue is placed superiorly and posteriorly to avoid interference and accidental injury during the procedure, a 1-cm midline incision can then be made through the mucosa halfway between the floor of the mandible and the submandibular ductal papillae. This incision can then be deepened by blunt dissection through three of the muscles of the floor of the mouth; the geniohyoid, genioglossus, and the anterior bellies of the digastric muscles. As depicted in Figure 2B, a curved hemostat can then be placed through the incision which is then used to grasp the pilot tube. This is done to create a continuous passage through the incision made [7].

**Figure 3:**
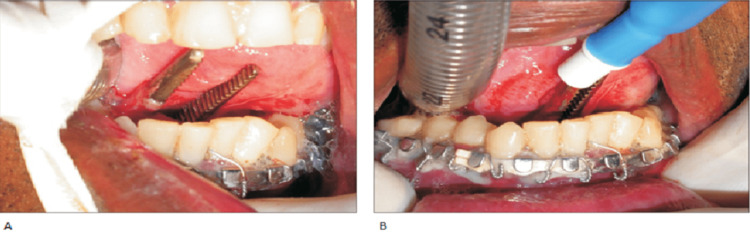
(A) Intraoral incision is made and a hemostat is utilized for the creation of continuity through the extraoral incision. (B) Pilot tube is grasped and pulled through the intraoral incision. Images obtained from The Korean Association of Oral and Maxillofacial Surgeons. Copyright © 2016 The Korean Association of Oral and Maxillofacial Surgeons. All rights reserved. License link (http://creativecommons.org/licenses/by-nc/4.0/) [7].

**Figure 4:**
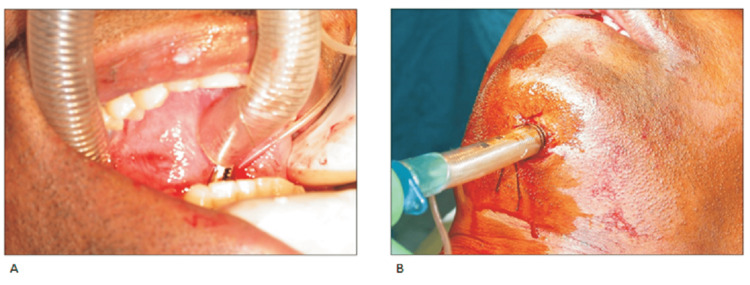
(A) The endotracheal tube is grasped and pulled through the submental region. (B) The endotracheal tube is stabilized with sutures. Images obtained from The Korean Association of Oral and Maxillofacial Surgeons. Copyright © 2016 The Korean Association of Oral and Maxillofacial Surgeons. All rights reserved. License link (http://creativecommons.org/licenses/by-nc/4.0/) [7].

As shown in Figures 3A, 3B, the hemostat functions to create a continuous passage where the pilot tube can be grasped and taken through the intraoral incision. After this is done the connector for the endotracheal tube can be removed and transferred externally via the submental incision made as depicted in Figure 4A [7].

The correct positioning of the Endotracheal tube in the trachea is confirmed using capnography and bilateral auscultation of the lungs, after which reconnection of the tube is done and sutures are used to stabilize the tube to the skin as shown in Figure 4B. This Submental technique allowed the endotracheal tube to be positioned above the mucosal floor of the mouth between the mandible and the tongue [7].

After reconstructive surgery is done the endotracheal tube and pilot tube can be re-passed through the passage created thereby achieving reversal of the technique. The incision can now be sutured and bandaged allowing proper wound healing [7]. Gonzalez-Magana et al. gave a summary of the steps taken which can be seen in Table 4 [25]. 

**Table 4:** Steps taken for the procedure of submental intubation Reprinted with permission from The Korean Association of Oral and Maxillofacial Surgeons [25].

Relevant steps	Reason/function
Markings	Surgical protocol for safe surgery
Intubation	Securing the airway
Releasing the connector of ETT from a fixed position	Avoids sudden maneuver after intubation
Incision	The posterior symphysis approach prevents damage to important anatomical structures
Dissection	Kocher forceps for a blunt dissection through skin, fat, platysma, anterior belly of the digastric muscle, mylohyoid muscles, and geniohyoid muscles and anterior to the submandibular glands and Warthon duct. To the floor of the mouth.
Passage of tube in Submental region	After releasing the connector, the pilot balloon and the endotracheal tube are passed sequentially through the channel.

**Figure 5:**
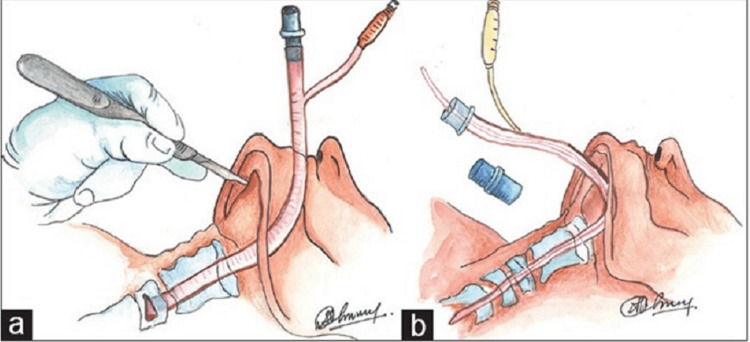
Graphical presentation of submental intubation. (a) Location of incision. (b) Repositioning of endotracheal tube. Reprinted with permission from Wolters Kluwer Medknow Publications, 2014. National Journal of maxillofacial surgery [26].

The location of the incision made during submental intubation can be seen in Figure 5A while Figure 5B shows the repositioning of the tube through the opening made [26].

Advantages of submental intubation 

Among the articles reviewed, it has been found that submental intubation was a preferred and more effective route than tracheostomy. It is a minimally invasive procedure that was easier to perform and could be completed in 10 minutes [9,15]. Tidke et al. stated that submental intubation can be achieved in the shortest time, the passage of the endotracheal tube and reattachment of the anesthetic circuit was achieved in 49.7 ± 24.8 seconds which is short enough without ventilatory support that the risk of hypoxia and its complications were low [13]. The duration of the procedure was anywhere between less than 4 to 30 minutes [27]. The procedure was not associated with an aesthetically unpleasing scar and was relatively inexpensive to perform. This method of intubation was associated with minimal alteration of the surrounding tissues for passage of the tube and there was complete dental occlusion which is important as maxillofacial trauma is often associated with dental disruption. Submental intubation provided unobstructed access to the surgical field and allowed both Surgeons and Anesthesiologists to perform their roles with minimal concerns for the interference of the endotracheal tube [8]. The increased postoperative care seen with a tracheostomy was not experienced when submental intubation was utilized, and the patients had a shortened hospital stay [9,11,13,15]. In one study, the tube was kept in place for 1 to 4 days and it was well tolerated. Due to this route of insertion of the tube and the type of tubed use, armored tubes, there arise less incidence of damage and kinking of the tube. It was also reported that the incidence of accidental biting of the tube was decreased compared to the oral endotracheal approach. The healing time after the procedure was an average of six days with a standard deviation of ±1.98 and the morbidity associated with the procedure was low. No mortality was reported [11].

Contraindications to the procedures mentioned were the need for long-term ventilatory support and maintenance, the presence of severe neurologic deficits, and patients with a history of keloid skin formation [13,19]. Submental intubation is well-favored, but it is not without its complications. It is not ideal in cases that require postoperative ventilatory support, or those that require multiple faciomaxillary surgical procedures [18,19]. There may be difficulties in suctioning as well as increases in airway pressures. As in all surgical procedures, there is a risk for infection, mucocele, fistula formation, and scarring that can occur at the site [15,20]. Excessive bleeding may occur intraoperatively and in the immediate postoperative period. There were increased risks of damage to surrounding structures such as the muscles, nerves, glands, and blood vessels [8,15]. Concerning the endotracheal tube, there may be damage, and premature extubation, thought of low incidence, may also occur. Amidst these possible complications, hardly any was reported in the articles reviewed. Gupta et al. stated that the only complication encountered during the procedure was that of damage to the pilot balloon of the Endotracheal tube which required a repeat procedure. [9,10]. One case was reported on a 30-year-old male who sustained trauma to the left jaw, experiencing increased peak airway pressures while undergoing the procedure. This was due to the endotracheal tube being compressed during its passage into the larynx, but this increase was not significant enough to convert the tube to an armored tube and risk loss of airway control [20]. Szantyr et al. reported that the incidence of complications varied from 0.24% to 7.13% in 842 of the patients gathered from 41 articles [10]. The success rate was reportedly 100% amongst the 842 patients [27]. Table 5 below depicts the incidence of submental intubation complications arising in 842 patients [10].

**Table 5:** Incidence of submental intubation complications Re-printed from Folia Medica Cracoviensia [10].

Complication	Patient number (% Incidence)
Infection	23 (2.7)
Fistula formation	10 1.1)
Endotracheal tube damage	10 (1.1)
Hypertrophic scar formation	3 (0.4)
Premature extubation	2 (0.3)
Excessive bleeding	2 (0.3)
Damage to the lingual nerve	1 (0.1)
Mucocele formation	1 (0.1)

Other complications reported were leakage and blockade of the tube along with damage to surrounding glands, e.g., submandibular and sublingual glands [15,25,27]. However, with excellent knowledge of anatomy and careful dissection, these complications become minimal and can be avoided altogether. It should also be mentioned that the period between transfer from an oral to submental intubation, and vice versa, may present with oxygen desaturation [10,13,15].

Limitations

The different techniques for submental intubation were not explored in this review. Improvements to the original techniques have been made over the years and more thorough coverage of new and improved techniques can be suggested by researchers. There is much room for increased exposure allowing for greater awareness of the method and utilization of submental intubation. The number of systematic review papers published on the topic was found to be few; researchers can aim to spread more knowledge and increase its popularity by increasing the number of systematic reviews being written. Another limitation faced in some cases reviewed is the time required for planning and preparation for the procedure which may have decreased its use in emergency settings. The articles reviewed had no reports of deaths associated with Submental Intubation therefore the incidence of mortality was not discussed.

## Conclusions

Submental intubation has been lauded to be a simple, easy technique for intubation when Orotracheal and Nasotracheal intubation is contraindicated. It has allowed excellent visualization and control of the operation field for surgeons and anesthesiologists as the intubation tube is not occupying the surgical field. Anesthesia for complex maxillofacial procedures and facial polytrauma patients can be achieved, both specialists can work comfortably and both roles can be carried out effectively with this technique. Among the articles reviewed, greater preference was for the submental intubation technique as it boasts minimal to no complications, a high compliance rate, and is ideal for patients who don't require long-term ventilation. Tracheostomy was utilized as a last resort due to its higher incidence of morbidity-associated and severe complications. Utilization of Tracheostomy was mostly seen in cases where there were contraindications to submental and nasotracheal intubation. Morbidity of Nasotracheal intubation was shown to increase in cases with skull base and naso-orbital-ethmoidal fractures; submental intubation can instead be considered.
